# Mutational profile of oropharyngeal squamous cell carcinoma in relation to HPV, tobacco smoking and prognosis with validation in the DAHANCA 19 randomized trial

**DOI:** 10.2340/1651-226X.2025.44042

**Published:** 2025-08-26

**Authors:** Jacob Kinggaard Lilja-Fischer, Morten Horsholt Kristensen, Pernille Lassen, Torben Steiniche, Trine Tramm, Magnus Stougaard, Anders Frederiksen, Benedicte Ulhøi, Jan Alsner, Kasper Toustrup, Christian Maare, Jørgen Johansen, Hanne Primdahl, Claus Andrup Kristensen, Maria Andersen, Jesper Grau Eriksen, Jens Overgaard

**Affiliations:** a Department of Experimental Clinical Oncology, Aarhus University Hospital, Aarhus, Denmark; b Department of Otolaryngology – Head and Neck Surgery, Aarhus University Hospital, Aarhus, Denmark; c Department of Clinical Medicine, Health, Aarhus University, Aarhus, Denmark; d Department of Oncology, Herlev Hospital, Herlev, Denmark; e Department of Pathology, Aarhus University Hospital, Aarhus, Denmark; f Department of Clinical Genetics, Aarhus University Hospital, Aarhus, Denmark; g Department of Oncology, Aarhus University Hospital, Aarhus, Denmark; h Department of Oncology, Odense University Hospital, Odense, Denmark; i Department of Oncology, Copenhagen University Hospital – Rigshospitalet, Copenhagen, Denmark; j Department of Oncology, Aalborg University Hospital, Aalborg, Denmark

**Keywords:** squamous cell carcinoma of head and neck, radiotherapy, human papillomavirus, biomarkers

## Abstract

**Background and purpose:**

This study investigated prognostic biomarkers in oropharyngeal squamous cell carcinoma (OPSCC), with a focus on tumors related to human papillomavirus (HPV) infection and potential molecular effects of tobacco smoking, as smokers with HPV+ OPSCC often have poorer outcomes.

**Patients/material and methods:**

We first analyzed 56 previously untreated OPSCC patients (exploration cohort), assessing HPV status, gene expression related to hypoxia, tumor subtype, and radiosensitivity, together with next-generation sequencing (NGS) of cancer-related genes. A custom NGS panel was subsequently designed and validated in 162 patients from the DAHANCA 19 randomized controlled trial (RCT), all treated with curative (chemo-)radiotherapy.

**Results:**

In the exploration cohort (40 HPV+, 79%), the most common molecular events in HPV+ tumors were PIK3CA and ATR mutations and chromosome 3q amplification. ATR and CREBBP mutations occurred more often in heavy smokers (>10 pack-years), but these associations were not confirmed in the DAHANCA 19 cohort. No specific smoking-related mutational signature or link to TP53 mutations was observed.

In the DAHANCA 19 cohort, 17 locoregional failures (LRF) occurred among 128 HPV+ patients. No unifying molecular features were identified. However, mutations in NFE2L2 and CASP8, as well as amplifications of 3q genes (BCL6, SOX2), were associated with LRF.

**Interpretation:**

In HPV+ OPSCC, only few molecular alterations appear to act as drivers or prognostic biomarkers. Importantly, no molecular features of tobacco smoking exposure were identified, and the mechanism behind the worse prognosis in smokers remains unclear.

## Introduction

The rapidly rising incidence of oropharyngeal squamous cell carcinoma (OPSCC) in developed countries has fortunately been accompanied by the recognition that Human papillomavirus (HPV), the causative agent, is also associated with a better prognosis compared to HPV-negative OPSCC [1–3]. On this basis, there has been significant interest in further personalizing treatment to reduce treatment induced morbidity in HPV+ OPSCC, and improve survival in HPV- OPSCC.

However, it was also noted that not all patients with HPV+ OPSCC had an equally favorable prognosis. Notably, Kian Ang and others observed that patients with a significant tobacco smoking history had a less favorable prognosis, which was also confirmed by Lassen et al. in an individual patient data meta-analysis [4, 5]. The exact mechanism has not been elucidated. Weinberger and others postulated that tobacco smoking induces additional mutations, most notably involving *TP53*, along with other molecular alterations that would impact disease biology and treatment response [6, 7].

Most of our biological knowledge of OPSCC comes from a few larger studies, especially the Cancer Genome Atlas (TCGA) study published in 2015, from which data have been further analyzed in numerous subsequent studies [8, 9]. However, these data are restricted by the limited number of OPSCC cases in the TCGA cohort as opposed to oral cancers, few HPV+ cases, heterogenous treatment, very few recurrences and inhomogeneous follow-up schedules. Thus, there is a need for further research in the molecular biology of OPSCC in the context of HPV and tobacco smoking with comprehensive outcome data in high-quality clinical cohorts.

The primary aim of this study was to characterize the molecular impact and mutational signature of HPV and tobacco smoking in a contemporary OPSCC cohort and to validate these findings in a larger cohort. The second aim was to assess the prognostic significance of these molecular alterations in HPV+ OPSCC treated with curatively intended (chemo-) radiotherapy in the DAHANCA 19 randomized, controlled trial. The hypothesis being that a distinct mutational signature can be identified in HPV+ OPSCC among tobacco smokers and that these alterations have a prognostic impact, which would essentially confirm the Weinberger hypothesis.

## Patients/material and methods

### Patients and treatment

Patients with previously untreated OPSCC who had consented to donation of tumor tissue were assembled in an exploration cohort and a validation cohort.

The exploration cohort consisted of patients treated at Aarhus University Hospital, and included in two prospective DAHANCA studies [10, 11]. All patients received curatively intended chemoradiotherapy according to DAHANCA guidelines.

The validation cohort consisted of a subcohort of patients from the DAHANCA 19 trial, which randomized 608 patients with squamous cell carcinoma of the pharynx, larynx and oral cavity to receive placebo or the monoclonal Epidermal growth factor receptor (EGFR)-antibody zalutumumab adjuvant to curatively intended (chemo-) radiotherapy (ClinicalTrials.gov identifier: NCT00496652; regional ethics committee approval no. 20070091) [12]. In this study we included only patients with primary tumor site in the oropharynx. Patients received 66–68 Gy in 33–34 fractions, 6 per week, with the hypoxic radiosensitizer nimorazole. Concurrent weekly low-dose cisplatin was prescribed for patients with stage III–IV disease. An alternative hyperfractionated schedule of 76 Gy in 56 fractions, 10 fractions per week without cisplatin, was permitted for patients with T2-4N0 tumors [13].

Patients were monitored until death or for a minimum of 5 years after completing treatment under a rigorous follow-up protocol at the treating institution. After 5 years, zalutumumab showed no effect on locoregional control or survival [12].

Patient, tumor and treatment characteristics were prospectively collected and available through the DAHANCA database [14]. Patients with a cumulative tobacco exposure of 10 or more pack-years were classified as tobacco smokers.

### DNA sequencing and analysis

In the exploration cohort we performed targeted sequencing of 409 cancer-related genes included in the Ion AmpliSeq Comprehensive Cancer Panel (CCP) using the Ion Torrent S5 platform running the Torrent Suite version 5.2.2 (Thermo Fisher Scientific). Results from this study as well as literature studies led to development of a smaller custom-made gene panel, which were utilized for the DAHANCA 19 validation cohort (Supplementary Table 1).

Briefly, formalin-fixed and paraffin embedded (FFPE) tumor samples were evaluated for tumor contents, and DNA isolated using a QIAsymphony SP and the QIAsymphony DSP DNA mini kit (ref. no: 937236). Library preparation (Ion AmpliSeq Library Kit 2.0, Thermo Fisher Scientific), amplification, and sequencing (using the Ion 540™ Kit-Chef and the Ion 540™ Chip Kit, Thermo Fisher Scientific) were performed according to the manufacturer’s instructions.

Variant calling was performed with Ion Reporter version 5.2 (Thermo Fisher Scientific) with an Ampliseq single sample workflow. Normal tissue control samples were used to exclude artifacts. To retain only non-synonymous variants with a probable somatic nature, we excluded common polymorphisms, sites with homopolymeres of more than 4 bases or less than 100 reads, homozygous variants and variants <10 reads or <3% of total reads. Variants still present were then presumed to be somatic mutations (Detailed in Supplementary Table 2). Detection of copy-number variation (CNV) was performed using the Ion Reporter CNV workflow.

### Quantitative Reverse transcription polymerase chain reaction (RT-PCR)

We used Quantitative PCR (qPCR) to quantify gene expression related to hypoxia, radiosensitivity, tumor subtype, immune infiltrate and cancer stem cell markers.

RNA was extracted from FFPE tumor tissue samples using an automated setup (Siemens Tissue Preparation System). Following complementary DNA (cDNA) synthesis, quantitative PCR was performed with TaqMan gene expression assays (Life Technologies). We assessed the expression of the 15 genes included in the hypoxia gene classifier described by Toustrup et al., and classified tumors as ‘more’ or ‘less’ hypoxic, as previously described [15]. Additionally, we examined the expression of 10 genes in the radiosensitivity index (RSI), which is the basis of the genome-based adjusted radiotherapy dose (GARD) concept proposed by Scott et al. [16]. Furthermore, we classified HPV-positive tumors into inflamed/mesenchymal (IMS-HPV) or classical (CL-HPV) subtypes based on the expression of a set of six genes previously identified by Keck et al. [17]. Finally, we analyzed the expression of *PD-1, PD-L1*, TRIP-12, MET, SLC3A2 and HPV16/18 E6 and E7 (See Supplementary Table 1).

### HPV classification

All tumors were examined for p16 expression by standard immunohistochemistry, and were classified as HPV+ according to published criteria [18]. Tumors in the exploration cohort were examined for HPV16 and HPV18 *E6* and *E7* RNA, with PCR primers designed in-house [19], or examined for high-risk HPV DNA with the INNO-LiPA® HPV Genotyping Extra II assay (Fujirebio). Patients in the DAHANCA 19 cohort was examined for presence of HPV DNA using a custom-designed HPV next-generation sequencing (NGS) panel, as previously reported [20].

### TCGA data extraction

To compare our data to an independent external cohort, we queried the TCGA Pan Cancer Atlas study at portal.gdc.cancer.gov and cbioportal.org [21]. From 523 HNSCC cases, 78 had positive oropharyngeal tumor site with matching clinical and molecular data, which were then downloaded. Data were analyzed both with the full gene set, and restricted to those in our custom gene panel.

### Statistics

Patient cohorts were characterized using descriptive statistics. Overall survival (OS) was analyzed as time from end of radiotherapy to death of any cause using Kaplan-Meier method, and compared with log-rank test. Locoregional failure (LRF) was analyzed as cumulative incidence, with death or distant metastasis considered competing events. Hazard ratios with 95% confidence intervals (CI) were calculated using Cox regression following confirmation of proportional hazard and the proportion of variation explained was estimated using Royston’s R². Correlation between molecular alterations and HPV status or LRF were analyzed using Fisher’s exact test, using false discovery rate corrected *q*-values to account for multiple comparisons. Gene expression levels were compared using rank-sum test due to non-normality. We considered *p*-/*q*-values < 0.05 to be statistically significant.

All statistical analyses were performed using Stata 14.2 (StataCorp, Texas, USA).

### Ethics

This study was approved by the Regional Health Ethics Committee (1-10-72-406-14) and reported to the Danish Data Protection Agency.

## Results

### Patient characteristics

Based on tumor site, tissue availability, and successful DNA sequencing, we included 56 patients with OPSCC in the exploration cohort and 162 patients in the DAHANCA 19 validation cohort (Supplementary Figure 1). Almost all patients had primaries of the tonsils or base of tongue, and 77% were HPV-positive (Table 1). More than half of patients with HPV-positive tumors were smokers, when defined as having a tobacco smoking history of 10 or more pack-years.

**Table 1:** Patient, tumor and treatment characteristics.

Characteristic	Exploration (*n* = 56)		DAHANCA 19 (*n* = 162)		TCGA (*n* = 78)	
**p16 status**						
Positive	40	71%	128	79%	48	62%
Negative	16	29%	34	21%	23	29%
Unknown					7	9%
**Age, years**						
Median (IQR)	59	54–66	58	53–64	56	49–62
**Gender**						
Male	41	73%	128	79%	69	88%
Female	15	27%	34	21%	9	12%
**Tumor site**						
Tonsil	41	73%	113	70%	42	54%
Base of tongue	14	25%	46	28%	25	32%
Oropharynx, other	1	2%	3	2%	11	14%
**T classification**						
T1	13	23%	39	24%	10	13%
T2	21	38%	75	46%	27	35%
T3	14	25%	36	22%	11	14%
T4	8	14%	12	7%	3	4%
Missing					27	35%
**N classification**						
N0	4	7%	25	15%	13	17%
N1	3	5%	28	17%	7	9%
N2	49	88%	101	62%	24	31%
N3	0	0%	8	5%	3	4%
Missing					31	40%
**Stage**						
I	1	2%	2	1%	3	4%
II	4	7%	14	9%	10	13%
III	4	7%	33	20%	9	12%
IV	47	84%	113	70%	24	31%
Missing					32	41%
**Smoking**						
<10 pack-years	21	38%	50	31%	7	9%
≥10 pack-years	35	63%	112	69%	36	46%
Missing					35	45%
Never	16	29%	32	20%		
Former	19	34%	86	53%		
Current	21	38%	44	27%		
**HPV and smoking combined**						
p16 positive <10 pack-years	21	38%	50	31%	4	5%
p16 positive ≥10 pack-years	19	34%	78	48%	22	28%
p16 negative	16	29%	34	21%	23	29%
Missing					29	37%
**Treatment**						
Radiotherapy, curative intent	55	98%	162	100%	54	69%
Radiotherapy, aborted	1	2%	0	0%	N/A	
Other/missing					24	31%
**Radiotherapy schedule**						
< 66Gy	1	2%	1	1%		
66Gy/33fx, 6/week	22	39%	87	54%		
68Gy/34fx, 6/week	31	55%	68	42%		
> 68Gy	2	4%	6	4%		
Missing					78	100%
**Chemotherapy**						
Yes	45	80%	136	84%		
No	11	20%	26	16%		
Missing					78	100%
**Radiosensitizer**						
Nimorazole	49	88%	160	99%		
Missing					78	100%
**Zalutumumab**						
Zalutumumab			80	49%		
Placebo			82	51%		
**No. of events at 5 years**						
Locoregional failure	7	13%	32	20%	N/A	
Death	18	32%	35	22%	20	26%
**Median follow up (years with IQR)**	5.1	5.0–5.3	5.2	5.0–11	2.1	1.5–3.5

Classification according to TNM7. IQR: Inter-quartile range.

### Mutations and CNV in the exploration cohort

The most frequently mutated genes among 40 HPV+ tumors were *SYNE1*, *KMT2C*, *DST* and *PIK3CA*, while *TP53* mutations were rare at just 5% (Supplementary Figure 2).

Taking both mutations and CNV in to account, *PIK3CA* and *ATR* were the most frequently altered genes among HPV+ tumors, while amplifications in genes located at chromosome 3q were also prominent. Also, deletions or mutations in *ATM* were notable (Supplementary Figure 3).

### Molecular alterations and tobacco in the exploration cohort

**Figure 1:**
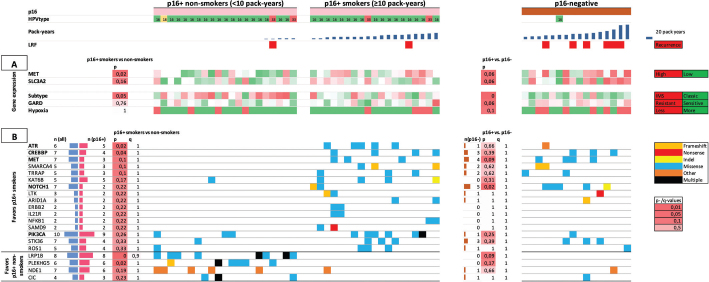
Fifty six patients of the exploration cohort in columns are arranged by p16-status and pack-years of tobacco smoking. A. Gene expression pattern of cancer stem cell markers (top rows); subtype, GARD and hypoxia (bottom rows). B. Top 20 genes (of 409) associated with smoking among p16+ patients. *P*- and *q*-values in the left column compare p16+ smokers vs. non-smokers, the right column compare p16+ vs. p16-negative. Most commonly mutated genes (horizontal) are arranged by association with smoking in p16+. Genes in bold were selected for inclusion in custom gene panel.

Figure 1 shows the molecular findings in the exploration cohort, with the HPV-positive cases ordered by increasing pack-years, HPV-negative cases are also shown for comparison.

Analysis of gene expression pattern among HPV+ tumors revealed significantly higher expression of the cancer stem cell marker *MET* among smokers (*p* = 0.02, Figure 1A). HPV-negative tumors also had higher levels of *MET* compared to HPV+ tumors (*p* = 0.06). The cancer stem cell marker SLC3A2 was also higher among smokers in HPV+ tumors, but not statistically significant (*p* = 0.16), and was also higher in HPV-negative than HPV+ (*p* = 0.06).

In HPV+ tumors, expression of genes related to the inflamed/mesenchymal subtype described by Keck et al. [17] was significantly higher among non-smokers vs. smokers (*p* = 0.05), while expression of genes related to RSI/GARD or the hypoxia-classifier was not associated with smoking.

Among HPV+ smokers, mutations in *ATR* and *CREBBP* were more frequent compared to non-smokers with *p* < 0.05 in univariate analysis, but this finding was not significant taking multiple comparisons into account (*q* = 1 for both, see figure 1B). Similarly, *LRP1B* and *PLEKHG5* were more frequently mutated among non-smokers, but with *q* > 0.9.

### Findings in the DAHANCA 19 validation cohort

The most frequently mutated genes in HPV+ tumors were *KMT2D* (65%), followed by *NOTCH1* (55%) and *EP300* (39%), see Supplementary Figure 4. Taking also CNV in to account, the most frequently altered gene in HPV+ tumors was ATM, with deletions or mutations affecting 84% of tumors (Supplementary Figure 5). In addition, amplifications or mutations in *KMT2D*, *TERT* and *NOTCH1* were also prominent.

Relating these findings to tobacco smoking in HPV+ patients, we found that *MET* mutations were more common among non-smokers (*p* = 0.003, *q* = 0.1). However, this is contrary to our findings in the exploration cohort, in which *MET* mutations were only found in smokers. Furthermore, the finding of an association between smoking and mutations in *ATR* and *CREBBP* could not be validated.

*TP53* mutations were common among HPV-negative patients in both cohorts (81 and 74%), but were rare among HPV+ patients (5 and 3%), with no evidence of any association with tobacco smoking. Gene expression analysis could not confirm elevated expression of MET in smokers and there was no difference in expression of hypoxia-related genes.

### Relation to prognosis in the DAHANCA 19 validation cohort

There were 17 LRF in 128 patients with HPV+ tumors in the DAHANCA 19 cohort. At 5 years, cumulative incidence of LRF was 11% (95% CI: 6–16%) in HPV+ patients, and 43% (95% CI: 25–61%) in HPV-negative. OS at 5 years was 88% (95% CI: 81–93) in HPV+ patients, vs. 41% (95% CI: 25–57%) in HPV-negative (Supplementary Figure 6).

In patients with HPV+ disease, we analyzed the prognostic value of age, gender, T and N classification, performance status, tobacco smoking status and accumulated pack-years. For tobacco smoking in HPV+ OPSCC, we found no prognostic effect of accumulated tobacco pack-years, but a trend toward worse OS and LRF in both former and current smokers. Based on calculated explained variation, these factors would account for less than half of the observed events, as detailed in Supplementary Table 3 and Supplementary Figure 7.

**Figure 2:**
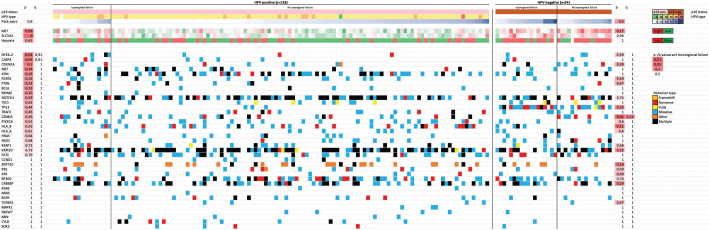
Patients in the DAHANCA 19 cohort (*n* = 162) grouped according to HPV-status and occurrence of locoregional failure. Top rows are HPV and tobacco pack-years. Middle rows are gene expression (Cancer stem cell markers and hypoxia) Bottom rows are genes, ranked according to *p*-/*q*-value for association with locoregional failure for HPV+ cases.

Figure 2 demonstrates mutations and CNV in the DAHANCA 19 validation cohort, stratified by HPV-status and grouped according to LRF or control. Mutations in *NFE2L2* and *CASP8* were associated with LRF in HPV+ patients (*p* < 0.05), but not when accounting for multiple comparisons (*q* > 0.9). Also, amplifications in genes located at 3q (*BCL6*, *SOX2*) were associated with LRF (*p* = 0.55) but not after multiple testing correction (*q* = 1).

### Comparison to TCGA cohort

Of the 78 patients in the TCGA cohort with known oropharyngeal tumor site, 71 had HPV-status, of which 48 were HPV-positive (See Table 1). Information on tobacco smoking exposure was available in 26 of 48 HPV-positive cases, which precludes a meaningful comparison to our findings.

Restricting the analysis to genes included in our custom gene panel, HPV+ tumors of the TCGA cohort was characterized by frequent mutations in the *PIK3CA* and *CYLD* genes, as well as amplification of genes at 3q and deletions of *ATM*, in concordance with our findings (Supplementary Figure 8). Mutations in *KMT2D*, *NOTCH1* and *EP300* were also frequent, but to a lesser extent than in the DAHANCA 19 cohort.

Relating molecular characteristics to prognosis (Progression-free survival) in the TCGA cohort, none of our findings in the DAHANCA 19 cohort could be confirmed, and vice versa (Supplementary Figure 9). However, this analysis was limited by the low number of OPSCC patients and few events in the TCGA cohort, which had a median follow up time of just 2.1 years.

## Discussion and conclusion

In this article we explored mutations and other alterations in HPV+ OPSCC, and sought to validate our findings in an independent, high-quality cohort. We show that tobacco smoking is not associated with specific mutations that could explain the increased risk of LRF and death reported in these patients. Furthermore, by integrating high-quality clinical and outcome data from a randomized trial with molecular tumor characterization, we aimed to more firmly assess the prognostic significance of molecular alterations. This study benefitted from the methodological rigor of a randomized clinical trial, which demonstrated that zalutumumab had no effect on prognosis, thereby rendering the cohort well-suited for the exploration of prognostic biomarkers. However, we did not identify any molecular alterations with prognostic impact. Notably, a substantial proportion of treatment failures remained unexplained by conventional clinical parameters.

Furthermore, the findings from the exploration cohort regarding the effect of tobacco smoking on mutational status could not be validated in the DAHANCA 19 cohort. This highlights the importance of validating results – particularly in studies involving extensive molecular data – an aspect that is all too often overlooked.

The past three decades have seen a dramatic rise in incidence of OPSCC, but our understanding of the disease biology, etiology and the most optimal treatment is still just evolving. In this study we demonstrate the molecular alterations common in OPSCC, with pronounced differences between HPV+ and HPV- tumors with regard to biology and prognosis, as shown previously [3, 8]. We also found a trend toward worse prognosis after curatively intended radiotherapy in former or current tobacco smokers with HPV+ OPSCC, in line with the observations by Ang et al., subsequently confirmed in meta-analyses [4, 5]. However, we could not identify any specific mutational pattern that could be attributed to the carcinogenic effects of tobacco smoking. These findings suggest that the mechanism behind the observed worse prognosis for patients with HPV+ OPSCC and a history of tobacco smoking is not through additional tumor tobacco-induced mutations. Notably, TP53 mutations were very rarely observed in HPV+ tumors and were absent even in heavy smokers, contrary to the Weinberger hypothesis [6]. This is consistent with observations in other studies [9, 22].

Other mechanisms must then be behind the observed worse prognosis in HPV+ OPSCC with a history of tobacco smoking. The MARCH-HPV meta-analysis demonstrated former or current smokers had increased T- and N-stage adjusted risk of LRF, which rules out comorbidity and other mortality. We can however not completely rule out a negative effect of smoking during radiotherapy in some patients. Still, there are suggestions of tobacco exposure modulating disease biology in these patients, possibly through modulation of the local tumor environment. For instance, tobacco smoking has been found to compromise oxygen delivery to tumors during radiotherapy [23]. Also, we have previously described tobacco exposure to be associated with significantly increased PD-L1 expression in HPV+ OPSCC, and others have consistently found increased presence of tumor-infiltrating lymphocytes [24–26]. This is an area of ongoing research.

Prognostic biomarker studies for HPV-positive OPSCC are particularly challenging due to the low incidence of disease recurrence in this patient population. This challenge was also evident in our study, where only 17 LRF events occurred among 128 patients. Despite available high-quality clinical data from a randomized controlled trial (RCT), we were unable to identify prognostic biomarkers. This challenge was further highlighted in our analysis of TCGA data, which included only 48 cases of HPV-positive OPSCC. Moreover, the TCGA dataset was characterized by inhomogeneous treatment and a lack of information on LRF, relying instead on progression-free survival as the endpoint, with a median follow-up time of only 2.1 years.

With these limitations in mind, it is clear that future studies on prognostic and predictive biomarkers for HPV+ OPSCC will require very large cohorts to obtain valid results. Some of this work is already ongoing, for instance in the PREDICTR-OPC project [27].

In summary, we have characterized the molecular landscape of OPSCC, which is largely determined by HPV. Tobacco smoking seems to be associated with worse prognosis in HPV+ OPSCC, but this effect is not driven by a particular mutational signature, contrary to previous hypotheses. Therapeutic outcome after radiotherapy in HPV+ OPSCC does not seem to be associated with specific molecular alterations.

## Supplementary Material



## Data Availability

The data that support the findings of this study are available from the corresponding author, JKL-F, upon reasonable request.
