# Supplementary material for: Mutational profile of oropharyngeal squamous cell carcinoma in relation to HPV, tobacco smoking and prognosis with validation in the DAHANCA 19 randomized trial
Source: Acta Oncol. 2025 Aug 26;64:44042. doi: 10.2340/1651-226X.2025.44042 (PMC12398107; doi:10.2340/1651-226X.2025.44042)

Supplementary material has been published as submitted. It has not been copyedited, or typeset by Acta Oncologica

Supplementary

Supplementary table 1. List of genes included in custom NGS panel (DAHANCA 19 cohort).

A. List of genes included in custom NGS panel (DAHANCA 19 cohort).

ATM, ATR, BCL6, CASP8, CCND1, CDKN2A, CDKN2B, CREBBP, CSMD3, CYLD, EGFR, EP300, FAT1, FBXW7, FGFR3, HLA-A, HLA-B, HRAS, KEAP1, KLLN, KMT2D, KRAS, MAPK1, MDM2, MET, NBN, NFE2L2, NOTCH1, NRAS, NSD1, PIK3CA, PTEN, RB1, SOX2, TBCD, TERC, TERT, TGFBR2, TP53, TRAF3, TSC1, ZNF750

B. List of genes included in RT-qPCR gene expression assays.

15-gene hypoxia classifier. (Toustrup et al)	*ADM, ALDOA, ANKRD37, BNIP3, BNIP3L, EGLN3, FAM162A, KCTD11, LOX, NDRG1, P4HA1, P4HA2, PDK1, PFKFB3, SLC2A1*
Gene-expression subtype. (Keck et al)	*ICOS, CD8A, LAG3, HLA-DRA, VIM, MMP9*
Radiosensitivity Index. (Eschrich et al)	*AR, JUN, STAT1, PRKCB, RELA, ABL1, SUMO1, PAK2, HDAC1, IRF1*
HPV (Schou et al)	*CDKN2A, HPV16: (E6, E7), HPV18: (E6, E7).*
Other (5)	*MET, SLC3A2, TRIP12, PDCD1* (PD-1), *CD274* (PD-L1)
Reference genes (Toustrup et al)	*ACTR3, NDFIP1, RPL37A*

Supplementary table 2.

NGS variant analysis. Variants were filtered in the Ion Reporter software using this filter chain to exclude artefacts or normal variants.

- 10 <= Alternate Allele Count <= 100000
- 0 <= Homopolymer Length <= 4
- Variant Effect in unknown, missense, nonframeshiftInsertion, nonframeshiftDeletion, nonframeshiftBlockSubstitution, nonsense, stoploss, frameshiftInsertion, frameshiftDeletion, frameshiftBlockSubstitution
- 0.02 <= Allele Ratio <= 1.0
- Location in splicesite_5, splicesite_3, exonic
- UCSC Common SNPs = Not In
- Variant Type in SNV, INDEL, MNV, CNV, LONGDEL, FUSION, ASSAYS_5P_3P
- Filtered Coverage >= 100
- <= Minor Allele Frequency <= 0.03
- <= 5000Exomes Global MAF <= 0.01
- Variant Classification in Pathogenic, Likely Pathogenic, VUS, Uncertain Significance, Unknown, Suspected Deleterious, Deleterious

Supplementary table 3. Univariate analysis of prognostic factors in HPV+ OPSCC patients in the DAHANCA 19 cohort (n=128).

		Locoregional failure		Overall survival	
	n	Events	HR (95% CI)	Event	HR (95% CI)
**All patients**	128	17		37	
**Age**					
<60	76	10	Ref.	16	Ref.
≥60 years	52	7	0.95 (0.36 - 2.5)	21	1.2 (0.59 - 2.2)
**Gender**					
Male	104	16	3.7 (0.49 - 28)	34	1.7 (0.52 - 5.7)
Female	24	1	Ref.	3	Ref.
**Performance status**					
0	114	13	Ref.	30	Ref.
1	13	4	2.6 (0.84 - 8.0)	7	2.2 (0.95 - 5.2)
2	1	0	N/A	0	N/A
**T**					
T1	32	3	Ref.	7	Ref.
T2	68	8	1.2 (0.32 - 4.5)	19	1.7 (0.71 - 4.2)
T3	20	3	1.5 (0.31 - 7.7)	6	2.6 (0.84 - 8.4)
T4	8	3	4.2 (0.85 - 21)	5	5.6 (1.7 - 19)
**N**					
N0	5	0	N/A	4	Ref.
N1	27	6	Ref.	12	0.57 (0.18 - 1.8)
N2	90	8	0.38 (0.13 - 1.1)	18	0.38 (0.12 - 1.2)
N3	6	3	2.7 (0.65 - 11)	3	1.8 (0.37 - 8.8)
**Tobacco status**					
Never	32	2	Ref.	2	Ref.
Former	74	10	2.1 (0.47 - 9.8)	27	2.6 (0.59 - 12)
Current	22	5	3.6 (0.70 - 19)	8	2.9 (0.58 - 14)
**Pack-years**					
<10	50	7	Ref.	8	Ref.
≥10	78	10	0.84 (0.32 - 2.2)	29	1.1 (0.50 - 2.6)

N/A: Not available.

Explained variation (R^2) for all variables combined in a multivariate model:
Locoregional failure: 0.33 (95% CI: 0.1 – 0.54).
Overall survival: 0.21 (95% CI: 0.04 – 0.41).

Supplementary figures.

Supplementary figure 1. Flowchart of included patients.
Cohorts are detailed in Lilja-Fischer (2019),[11] Toustrup (2016)[10] and Eriksen (2018).[12]

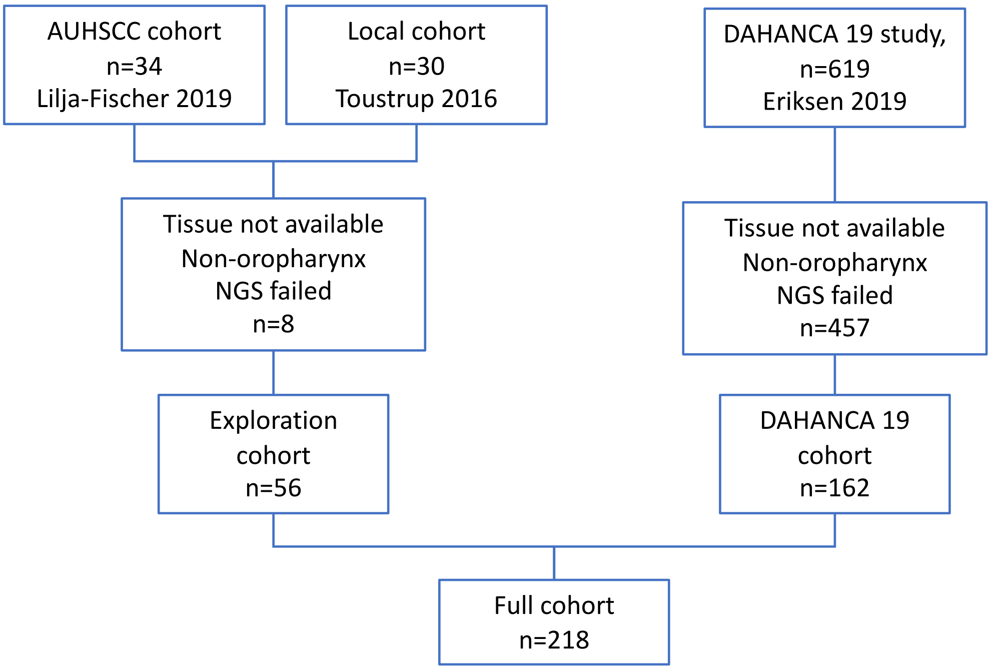

Supplementary figure 2. Top mutated genes (rows) from the CCP panel in 56 OPSCC tumors (columns). Top half shows tumors most frequently mutated, bottom half genes that were also selected for a custom gene panel. Genes are ordered by frequency among p16+ tumors.

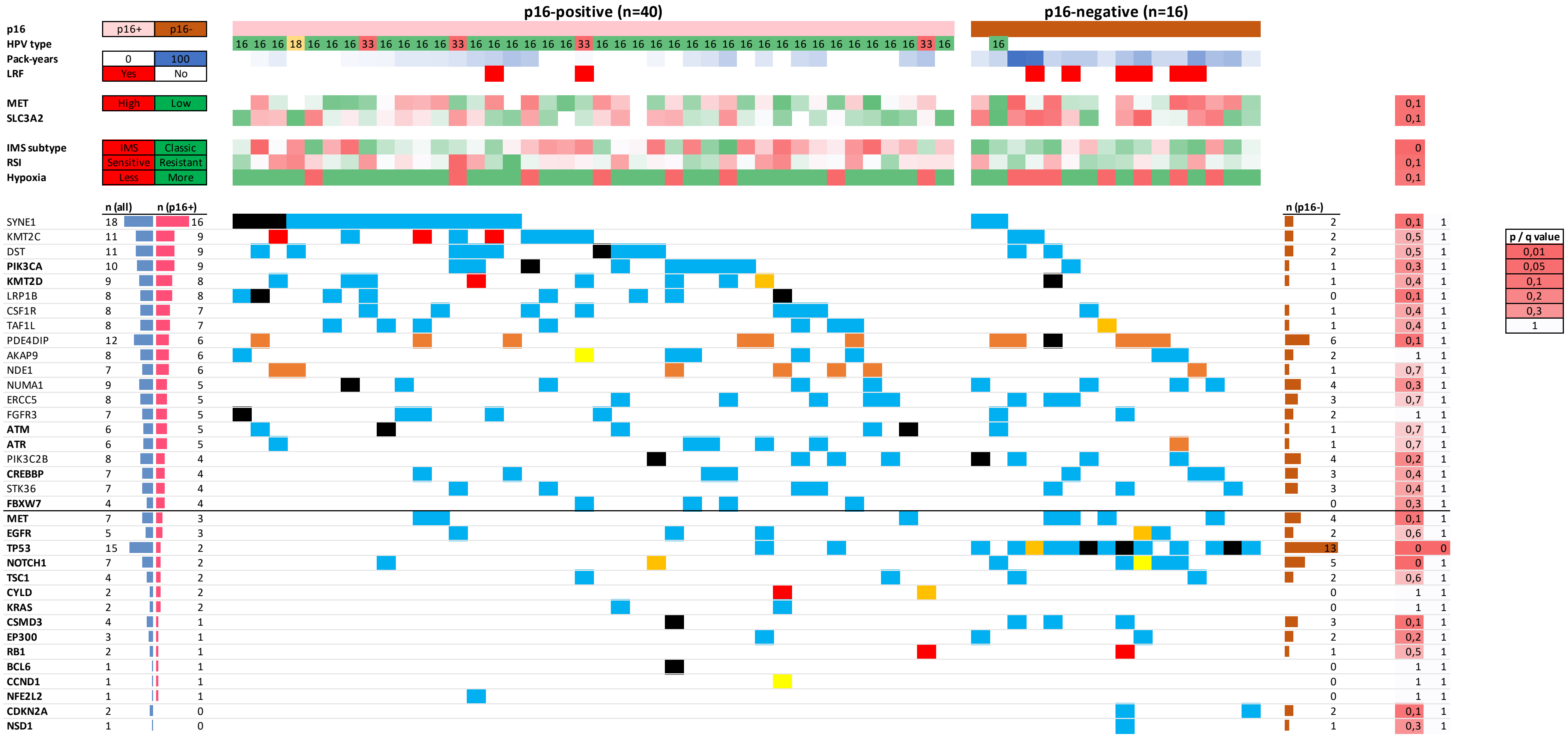

Supplementary figure 3. Top altered (mutation, amplification, or deletion) genes in rows from the CCP panel in 56 OPSCC tumors (columns). Top half shows genes most frequently altered, bottom half shows genes that were also selected for a custom gene panel. Genes are ordered by frequency among p16+ tumors.

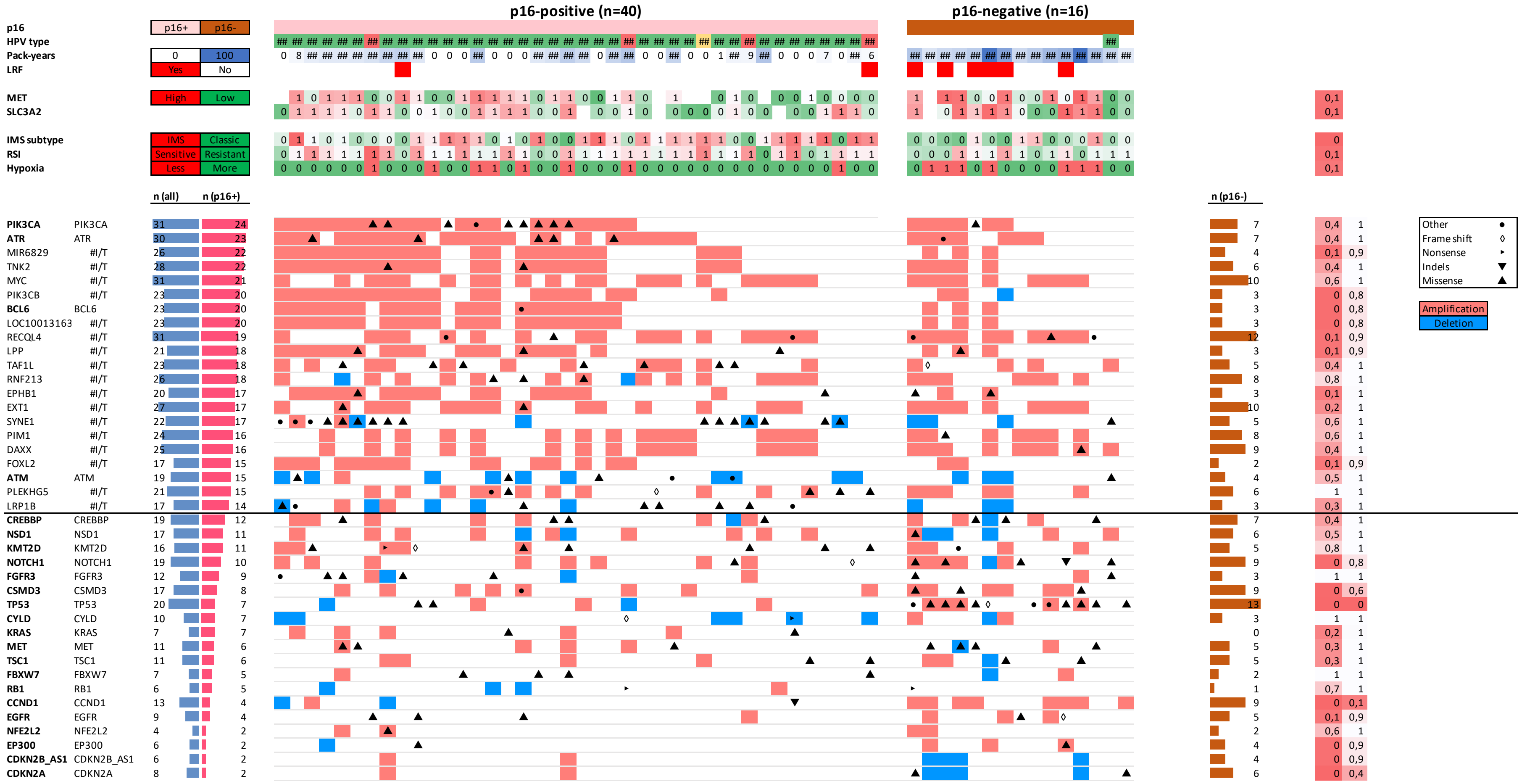

Supplementary figure 4. Top mutated genes in a custom gene panel in the DAHANCA 19 cohort.

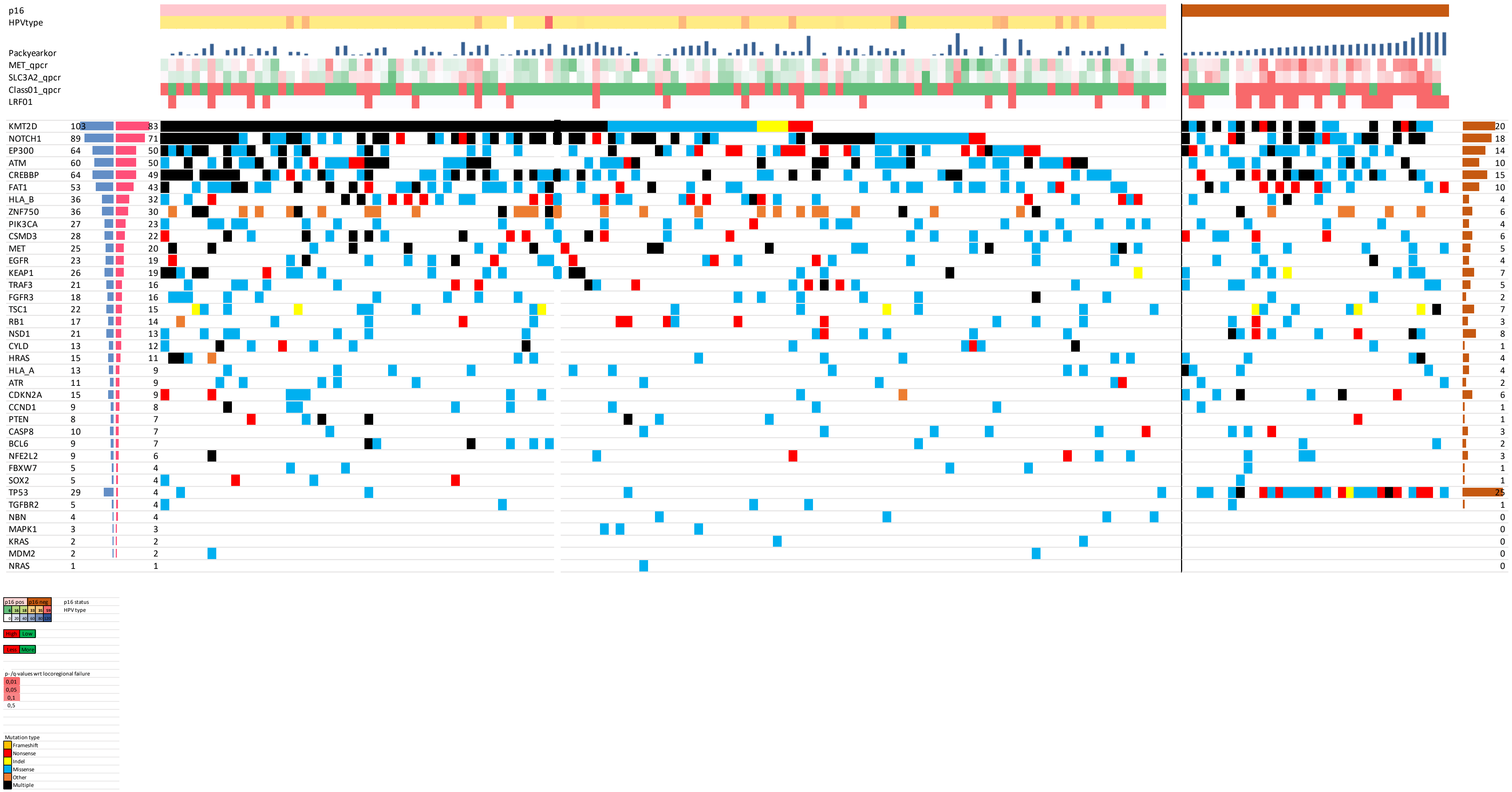

Supplementary Figure 5. Mutations and CNV in the DAHANCA 19 cohort (n=162) characterized with a custom NGS gene panel.

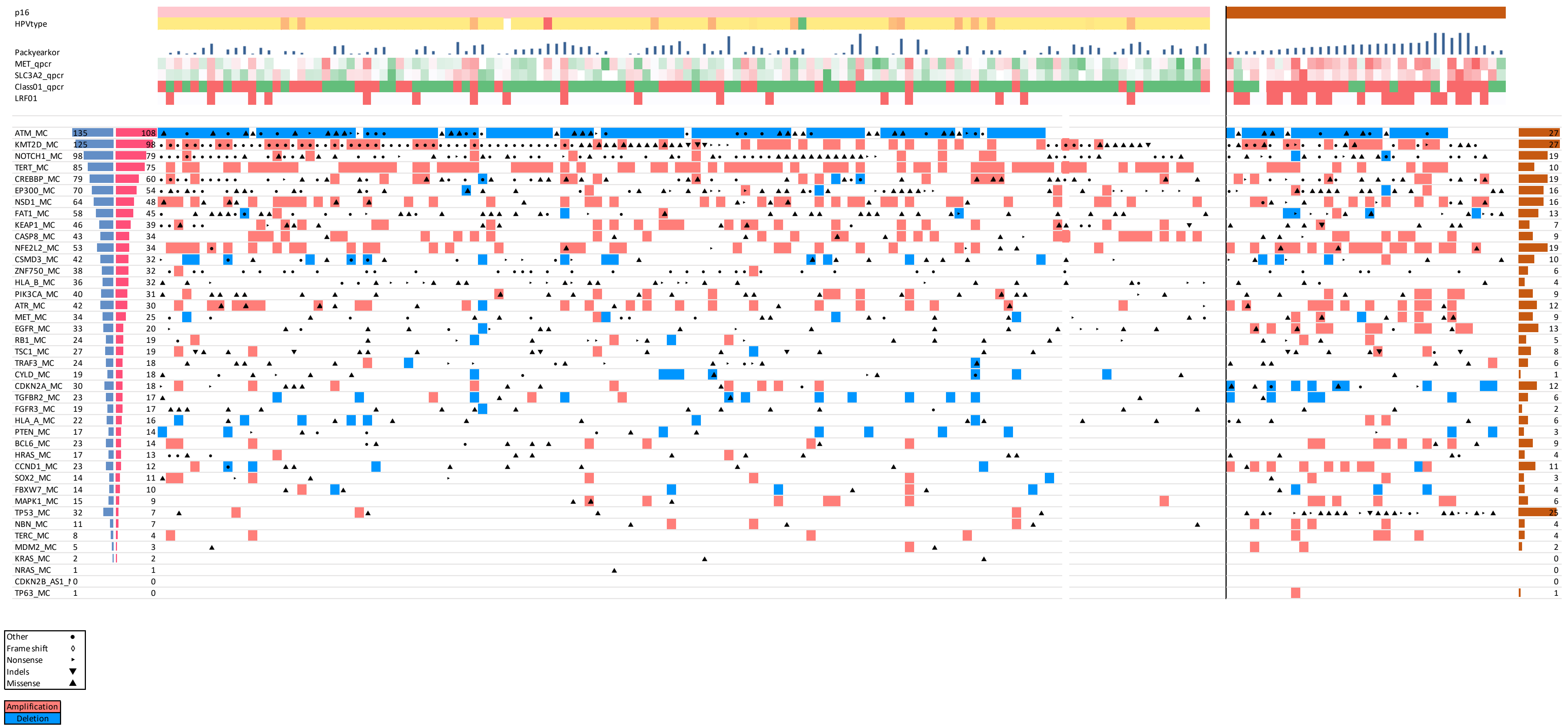

Supplementary figure 6. Overall survival and locoregional failure in the DAHANCA 19 cohort by HPV-status.

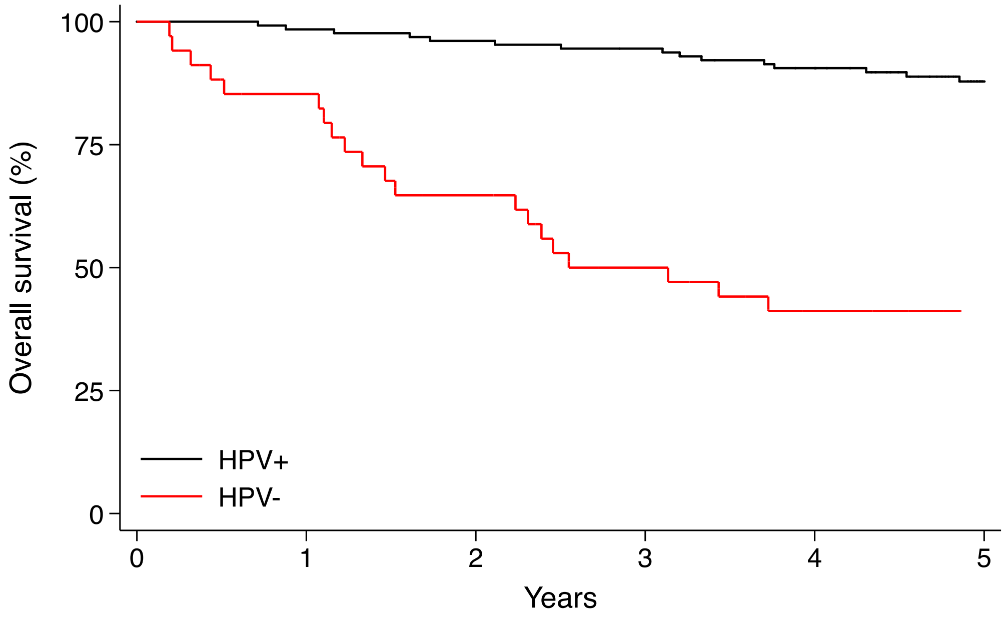

Number at risk

HPV+	128	126	123	120	112	76
HPV-	34	29	22	17	14	12

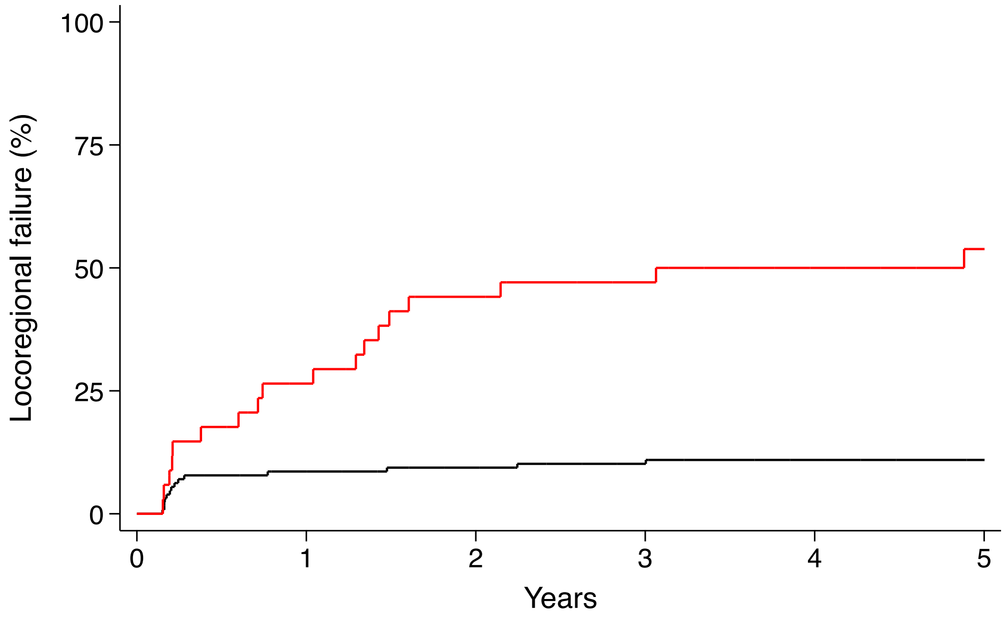

Number at risk

HPV+	128	117	113	108	104	69
HPV-	34	23	16	15	13	9

Supplementary figure 7. Prognostic factors for locoregional failure and overall survival for HPV+ patients in the DAHANCA 19 cohort.

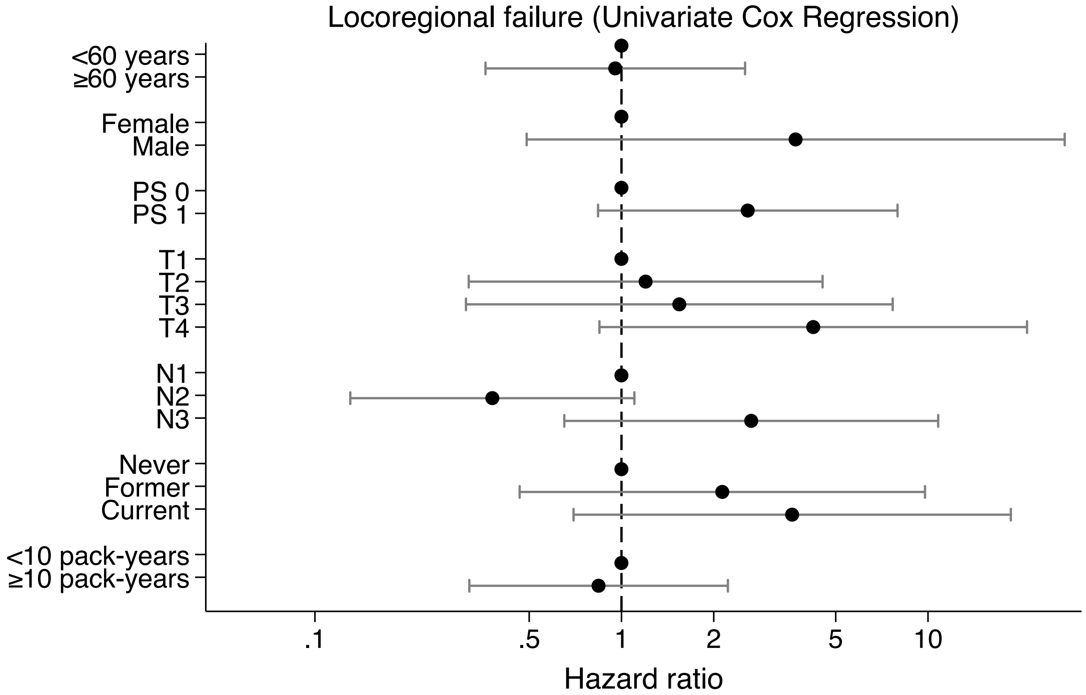

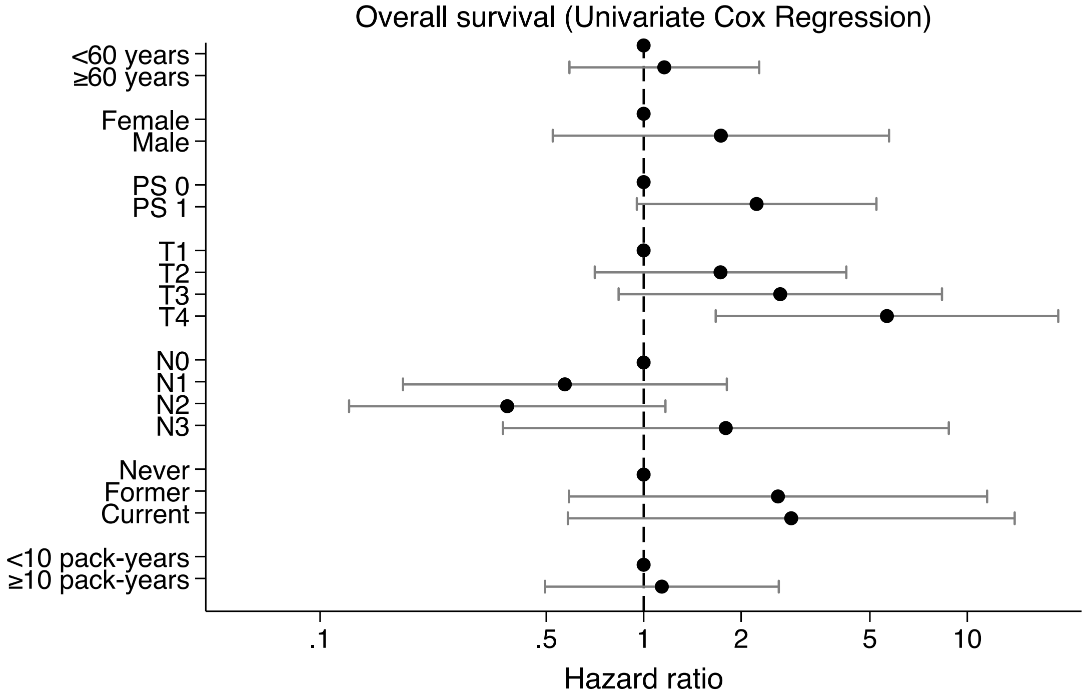

Supplementary figure 8. Distribution of mutations (top) and CNV (bottom) in HNSCC patients in the TCGA Pan Cancer Atlas with oropharynx tumor site, restricted to genes included in our custom gene panel.

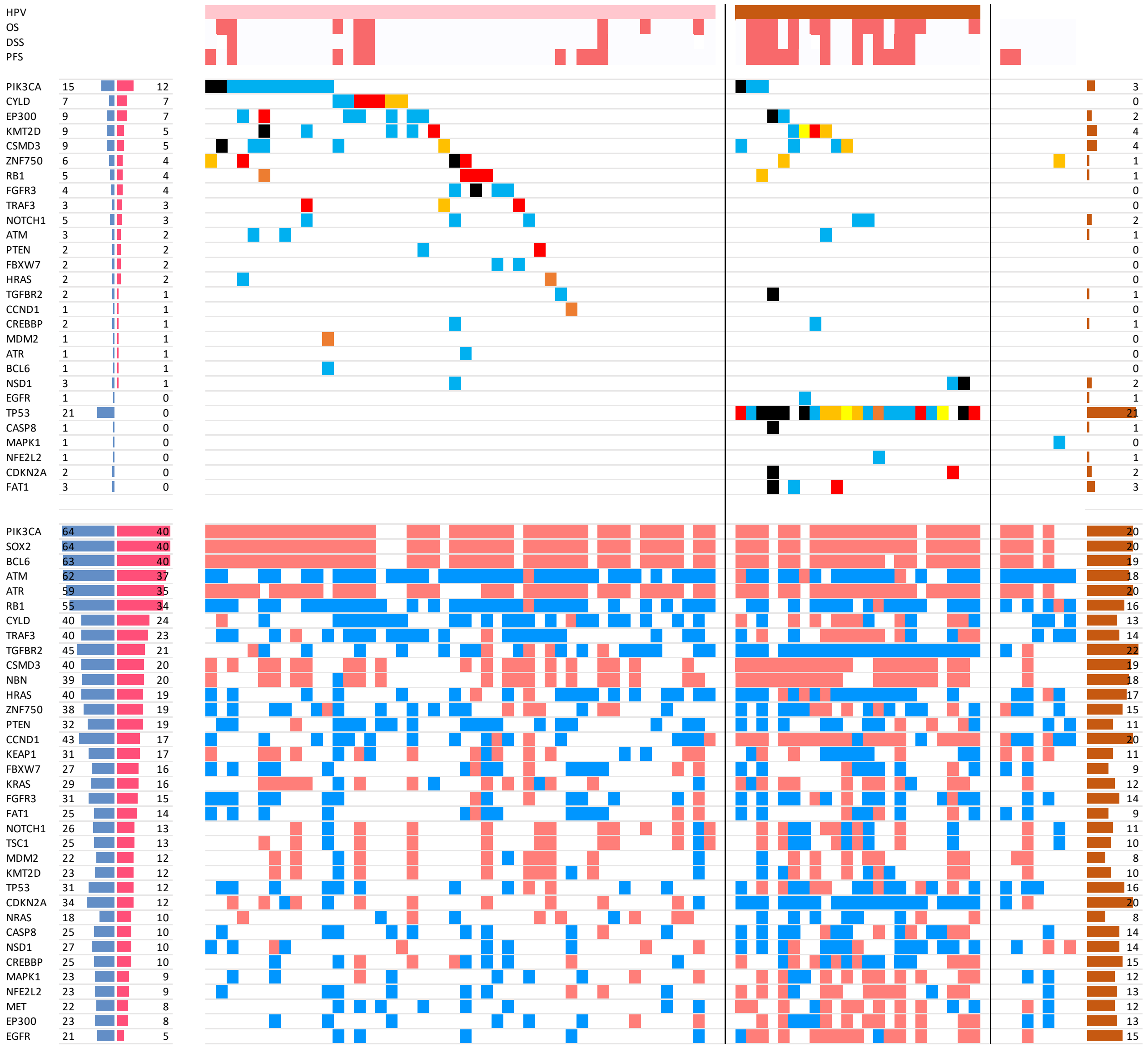

Supplementary figure 9. Mutations (top) and CNV (bottom) of OPSCC in the TCGA Pan Cancer Atlas cohort. Cases are ordered by HPV-status and PFS-status. Genes are ordered by p-value for association with PFS within each category.

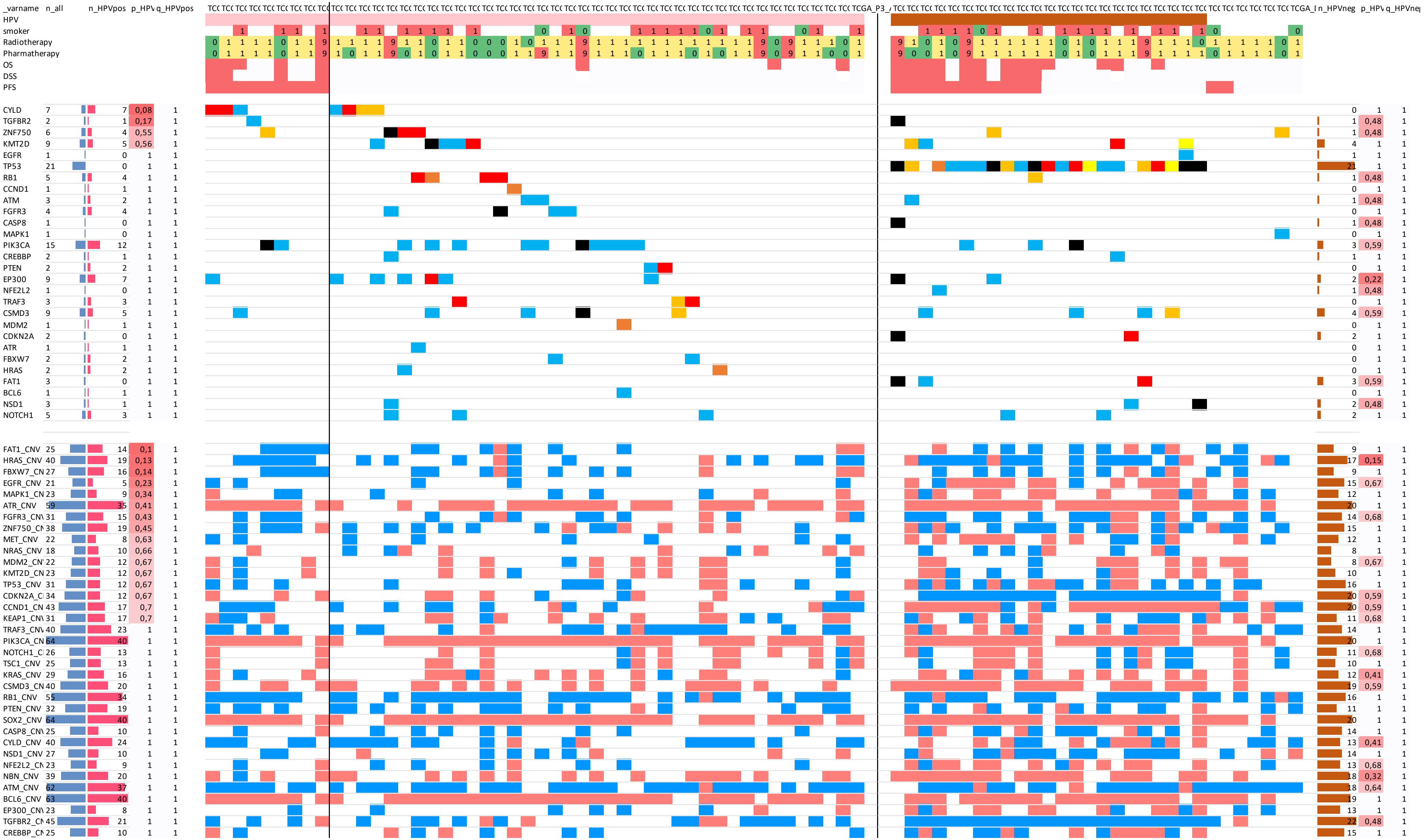

Supplement: Supplementary file 1 [file AO-64-44042-s1.pdf]
